# Assessment of the Safety of Tofacitinib Among Patients With Psoriasis: A Systematic Review and Meta-Analysis

**DOI:** 10.7759/cureus.70196

**Published:** 2024-09-25

**Authors:** Saad Alqahtani, Samia Khalil, Basel H Bakhamees, Layan M Almutairi, Mariyah A Alyahya, Waleed Khalid Z Alghuyaythat, Fatimah K Alabdulqader, Dana Aldossary, Shuruq Talea B Asiri, Talal A Alsulami, Abdulrahman A Hussain, Mohammed A Alaamer

**Affiliations:** 1 Family Medicine, King Salman Armed Forces Hospital, Tabuk, SAU; 2 Medicine and Surgery, Ibn Sina National College for Medical Studies, Jeddah, SAU; 3 Faculty of Medicine, King Abdulaziz University, Jeddah, SAU; 4 College of Medicine, Princess Nourah Bint Abdulrahman University, Riyadh, SAU; 5 Faculty of Medicine, University of Tabuk, Tabuk, SAU; 6 College of Medicine, Majmaah University, Al Majma'ah, SAU; 7 General Practice, King Fahad Hospital-Al-Hofuf, Al Hofuf, SAU; 8 Medicine and Surgery, Imam Abdulrahman Bin Faisal University, Dammam, SAU; 9 Facility of Medicine, Najran University, Najran, SAU; 10 General Practice, University of Jeddah, Jeddah, SAU; 11 General Practice, King Saud Bin Abdulaziz University for Health Sciences, Riyadh, SAU; 12 Collage of Medicine, King Saud Bin Abdulaziz University for Health Sciences, Riyadh, SAU

**Keywords:** herpes zoster, infections, nasopharyngitis, psoriasis, safety, tofacitinib, upper respiratory tract

## Abstract

Psoriasis is a clinically heterogeneous, lifelong skin condition. Novel medications such as Janus kinase (JAK) inhibitors have emerged as a promising class of agents that modulate the immune response. Tofacitinib could reduce skin lesions and boost patient-reported clinical outcomes, but its immunosuppressive effects might also increase patients' risk of infections and other adverse effects. To assess the safety outcomes of using Tofacitinib in comparison to placebo in terms of the incidence of serious infections, herpes zoster infection (HZ), upper respiratory tract infections (URTI), nasopharyngitis, and serious adverse events (SAE), this systematic review and meta-analysis followed the Cochrane Handbook for Systematic Reviews and Preferred Reporting Items for Systematic Reviews and Meta-Analyses (PRISMA) guidelines. The search strategy involved five databases. Two authors independently screened and selected studies. Only randomized controlled trials (RCTs) were eligible for inclusion. Data on baseline patient characteristics and outcomes such as serious infections, HZ infections, nasopharyngitis, and URTIs were extracted. The risk of bias was assessed using the revised version of the Cochrane risk of bias (ROB2) tool, and meta-analysis was conducted using Cochrane's Revman web. The OR was employed to estimate outcomes. A probability value of less than 0.05 was considered statistically significant. The search strategy initially identified 998 studies; of these, six RCTs were included with a total of 2516 participants having moderate-to-severe psoriasis and treated with either Tofacitinib or placebo. The meta-analysis results revealed increases in the risks of HZ, URTIs, serious infections, nasopharyngitis, and SAE, but no significant difference was found between the intervention and placebo groups. The risk of bias was assessed using the ROB2 tool, revealing low bias across most domains, with some concerns in certain studies related to deviations from intended interventions and missing outcome data. In conclusion, while Tofacitinib is effective against moderate-to-severe psoriasis with non-significant risks, there are some safety concerns regarding the measured outcomes. Future research is needed concerning the assessment of safety outcomes with long-term use of Tofacitinib among larger populations.

## Introduction and background

Psoriasis is a clinically heterogeneous, lifelong skin condition that presents in multiple forms. It has been described as a chronic inflammatory skin condition associated with increased proliferation of keratinocytes and an immune response. It is a major human pathologic condition that affects not only the physical and mental status of the patient but also their quality of life. The prevalence of psoriasis accounts for 0.33%-0.6% across different races and impacts about 125 million inhabitants of the Earth [1]. Psoriasis is characterized by the dysregulation of the immune system where innate and adaptive immunity are triggered by cytokines such as TNF-α, IL-17, and IL-23 [2].

Treatments for psoriasis include topical agents such as vitamin D analogs and corticosteroids, phototherapy, standard systemic treatments including methotrexate and ciclosporin, small molecule inhibitors, and biologic therapies. The advances in pharmacotherapies have paved the path toward the development of effective and targeted medications [3].

Recently, the discovery of targeted immunotherapies has changed the approaches to treating psoriasis. New therapies have been developed for moderate to severe disease cases [4].

Some patients may not respond to conventional therapy, which poses the need for alternative medications. Among these novel medications, Janus kinase (JAK) inhibitors have emerged as a promising class of agents that modulate the signaling pathways integrated into the immune response. JAK inhibitors, a type of small molecule targeted synthetic disease-modifying antirheumatic drug (DMARD), suppress the JAK/Signal Transducer and Activator of Transcription (STAT) pathway, inhibiting the upregulation of pro-inflammatory genes involved in articular and extraarticular inflammation [5]. One of these medications, Tofacitinib, a JAK inhibitor, has been studied for its efficacy and safety in managing psoriasis and psoriatic arthritis [6,7].

Many clinical trials have suggested that the drug Tofacitinib could reduce skin lesions and boost patient-reported clinical outcomes, but its immunosuppressive effects might also mean patients are at increased risk of infections and other adverse effects [8,9].

Even though Tofacitinib is an effective therapy for psoriasis, the literature clearly shows that its safety profile needs careful consideration; importantly, psoriasis itself is a systemic inflammatory disease that comes with many comorbidities, including an increased risk of cardiovascular cases, metabolic syndrome, and infections. Thus, adding JAK inhibitors such as Tofacitinib may increase the burden on patients by increasing the risk of infections or other health complications due to the suppression of immune responses, as well as causing side effects associated with the inhibition of signaling pathways [6]. For this reason, these medications need to be monitored for adverse effects, including infections, malignancies, and other complications related to immunosuppression. Serious adverse events (SAE) highlight the need for a personalized approach to treatment and may revolve around weighing the benefits of Tofacitinib against each patient’s risk factors and comorbid diseases [8].

Moreover, the long-term safety of Tofacitinib is still unclear. It is worth noting that psoriasis is a chronic condition requiring sustained therapy over a long period. Thus, understanding the long-term implications of Tofacitinib therapy is significant. Previous studies have explored the effects of continuous versus intermittent treatment regimens, focusing on issues such as drug withdrawal, retreatment, and the sustainability of clinical benefits over time [10].

Therefore, we conducted this systematic review and meta-analysis to assess the safety outcomes of using Tofacitinib in comparison to placebo in terms of the incidence of serious infections, SAE, herpes zoster infection (HZ), upper respiratory tract infections (URTI), and nasopharyngitis.

## Review

Methodology

The study was conducted following the principles of the Cochrane Handbook for Systematic Reviews of Interventions, version 6, and reported according to the Preferred Reporting Items for Systematic Reviews and Meta-Analyses (PRISMA) guidelines [11].

Eligibility criteria

Types of Studies Included

We included only randomized clinical trials (RCTs) published in English.

Participants

Studies that included adult patients with moderate-to-severe psoriasis.

Interventions

Direct comparison between the administration of Tofacitinib versus placebo.

Exclusion criteria

We excluded conference abstracts, duplicate reports, case reports, observational studies, review articles, editorials, clinical guidelines, and studies involving patients with other forms of psoriasis or with no clear reported outcomes.

Search strategy

An online search was performed using five databases: PubMed, Medline, Cochrane (CENTRAL), Web of Science, Ovid, and Scopus. No search filters were used, and the search encompassed the period from inception until August 2024. The English keywords used in this study were “Psoriasis” and “Tofacitinib.” Articles from the reference lists of relevant studies were also manually searched and reviewed.

Selection of studies

The processes of online searching, screening titles and abstracts, as well as reviewing the full texts of relevant articles, were conducted by two authors. Any disagreements were resolved by consensus.

Data extraction

Two authors independently extracted and recorded the following data: study ID (author and year of publication), study design, baseline patient characteristics (sample size, age, sex, Psoriasis Area and Severity Index Score (PASI), Body Surface Area (BSA%), Physician's Global Assessment (PGA), and Dermatology Life Quality Index Scores (DLQI)), and outcomes of interest such as the number of serious infections, HZ infections, nasopharyngitis, and upper respiratory tract infections.

Measured outcomes

Primary Outcomes

The incidence of serious infections, HZ, URTI, and nasopharyngitis.

Secondary Outcomes

The incidence of SAE.

Assessment of the risk of bias (ROB) in the included studies

We assessed the ROB using the ROB2 tool for RCTs, as all included studies were RCTs [12]. The ROB2 tool comprises five domains: randomization, deviations from the assigned treatment, missing data, measurement of the outcome, and selective reporting of outcomes and results. Moreover, the overall ROB is assessed by selecting the highest level of ROB from the five domains. The figures were visualized using the Robvis tool [13].

Statistical analysis

The meta-analysis was conducted using the Cochrane Revman web version 5. The pooled ORs with 95% CIs were measured for the following types of infections: HZ infections, URTIs, serious infections, nasopharyngitis, and SAE. Serious infections were classified as those requiring intravenous antimicrobial therapy, hospitalization for treatment, or meeting other criteria that justified such classification. Heterogeneity was measured across studies in the pooled analysis using the I2 statistics, where an I2 greater than 50% indicated substantial heterogeneity. If substantial heterogeneity was found, a random-effects model was applied to conduct the meta-analysis of the pooled data. A probability value less than 0.05 was considered statistically significant.

Results

**Figure 1:**
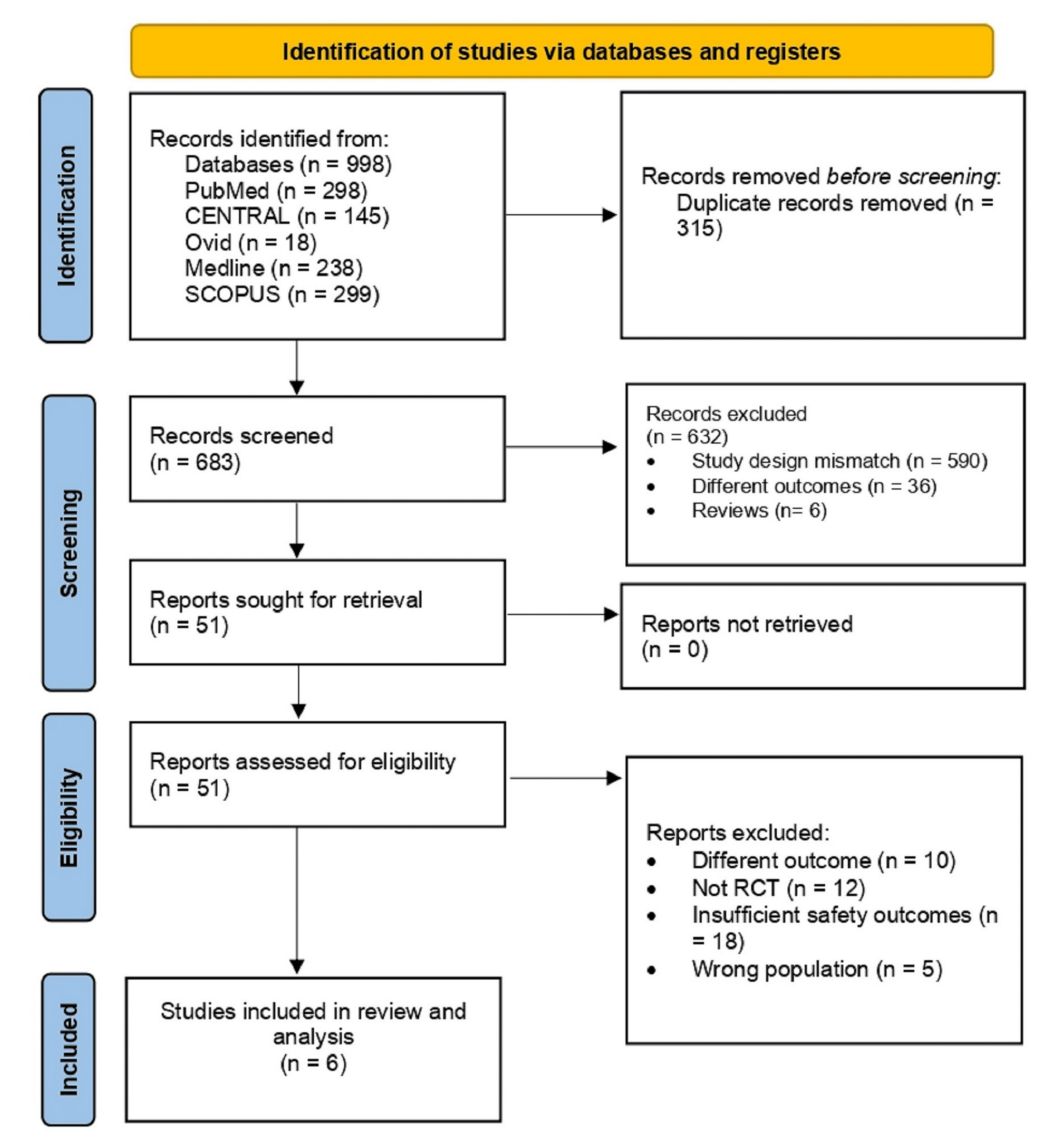
PRISMA flowchart of the selection process. PRISMA: Preferred Reporting Items for Systematic Reviews and Meta-Analyses.

The search strategy yielded 998 studies, out of which 315 were duplicates. The remaining 683 studies underwent screening of their titles and abstracts, and 632 records were excluded as follows: study design mismatch (n = 590), different outcomes (n = 36), and reviews (n = 6). The full texts of the remaining 51 studies were obtained and assessed for eligibility. Out of them, six RCTs were finally included in the review [14,15,16,17,18]. Figure 1 shows the PRISMA flow chart detailing the selection process of the included studies.

The major characteristics of the included studies

All the included studies were RCTs. One study was conducted in Japan [14], one in China [15], one in France [16], and three trials in the USA [17, 18]. All studies involved patients with moderate-to-severe psoriasis, treated with either oral or topical Tofacitinib compared to placebo. The review included 2,516 patients with psoriasis. The median age of the participants ranged from 40 to 46 years old. The baseline characteristics of the participants are summarized in Table 1.

**Table 1:** Summary of the major characteristics of the included studies. PASI: Psoriasis Area and Severity Index score; BSA: % Body Surface Area; PGA: Physician's Global Assessment; DLQI: Dermatology Life Quality Index score. (*) The number of psoriasis and placebo participants is counted from only the Tofacitinib and placebo groups, respectively. (**) The number of males (%) and baseline characteristics are counted from all participants across all treatment groups.

Study authors	Intervention	Study Design	Number of Participants (Psoriasis)^*^	Number of Participants (placebo)*	Age (Mean, SD) or (Median, IQR)	Male, n (%) **	Baseline Mean or median PASI score, mean or median % Body surface area (BSA), PGA (physician's global assessment) (n for both moderate and severe categories, %), and DLQI score (mean, SD)
Abe M et al. (2017) [14]	Tofacitinib (oral)	Phase 3 placebo-controlled RCT	46	12	Median age (IQR): 50.0 years (Q1 40.0, Q3 58.0)	48 (82.8%)	Median PASI score of 22.8 (Q1 19.5, Q3 36.9); Median % BSA = 37.0 (Q1 28.0, Q3 57.0); PGA of moderate = 53 (91.4); PGA of severe = 5 (8.6); Median DLQI of 8.0 (Q1 4.0, Q3 12.0)
Zhang J et al. (2017) [15]	Tofacitinib (topical)	Phase 3 placebo-controlled RCT	178	88	Mean (SD): 41.1 (12.3)	194 (72.9%)	Mean PASI score of 25.6 (±9.6 SD); mean % BSA =36.5% (±18.2 SD); PGA of moderate = 221 (83.1); PGA of severe = 45 (16.9); DLQI of 13.3 (7.1)
Bachelez H et al. (2015) [16]	Tofacitinib (oral)	Phase 3 randomized, double-blinded, double-dummy, placebo-controlled, and active comparator-controlled trial (double-blinded, phase 3 POETYK PSO-1 trial)	659	107	Median (range): For 5 mg Tofacitinib = 44·0 (18–73); for 10 mg Tofacitinib = 44·0 (19–75)	778 (71.0%)	PGA of moderate = 898 (81.6%); PGA of severe = 186 (16.9%)
Papp KA et al. (2015) (Trial a) [17]	Tofacitinib (oral)	Phase-2, randomized, double-blind, placebo-controlled, dose-escalation study	723	177	Median (range) = For 5 mg = 46.0 (18–78); for 10 mg = 46.0 (18–79)	643 (71.4%)	Median PASI score of 19.5/ 20.4/ 19.8; Median % BSA = 24/ 26.5/ 25.0; PGA moderate = 809 (89.9%); PGA severe = 89 (9.9%)
Papp KA et al. (2015) (Trial b) [17]	Tofacitinib (oral)	Phase-2, randomized, double-blind, placebo-controlled, dose-escalation study	763	196	Median (range) = For 5 mg = 47.0 (19–79); For 10 mg = 44.0 (18–82)	268 (70.2%)	Median PASI score = 20.7/ 19.3/ 20.1; Median% BSA = 26/ 24/ 23.6; PGA moderate = 784 (81.8%); PGA severe = 173 (18%)
Papp KA et al. (2012) [18]	Tofacitinib (oral)	Phase 3, randomized, multicentre, double-dummy, placebo-controlled, non-inferiority trial	147	50	Mean (SD) for 2 mg = 45.7 (1.8); 5 mg = 44; (12.6); 15 mg = 43.6 (15.6)	125 (63.5%)	Mean PASI score of 21.7; Mean % BSA = 30.4; PGA moderate = 146 (74.1%); PGA severe = 16 (8.1%)

Meta-analysis

Since the studies were conducted in various locations with different population sample sizes, we used a random-effects meta-analysis with ORs to assess safety outcomes among the included studies. The outcomes evaluated were the risk of serious infections, HZ, URTIs, nasopharyngitis, and SAE. We assessed heterogeneity using the I^2^ statistic. Since the number of studies was less than ten, we did not measure publication bias.

The risk of serious infections

**Figure 2:**
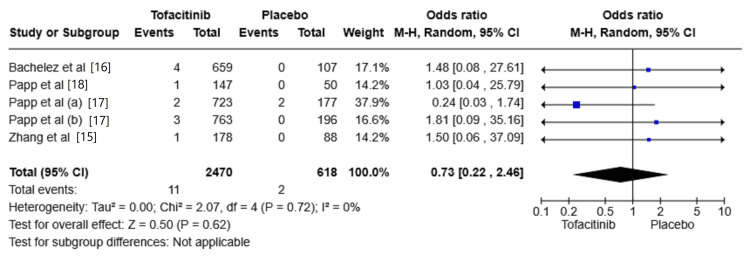
Random-effects meta-analysis from studies assessing the risk of serious infections. CI: Confidence Interval; df: Degrees of Freedom; OR: Odds Ratio; Z: Z-score; Chi²: Chi-square Test. The P-value is considered significant at (P < 0.05). Source: References [15-18]

Five studies reported the incidence of serious infections [15-18]. The risk of serious infections was increased in the Tofacitinib group compared with placebo (OR: 0.73, 95% CI: 0.22-2.46; I² = 0%); however, the results showed no significant difference between the two groups (P = 0.62) (Figure 2).

The risk of HZ infections

**Figure 3:**
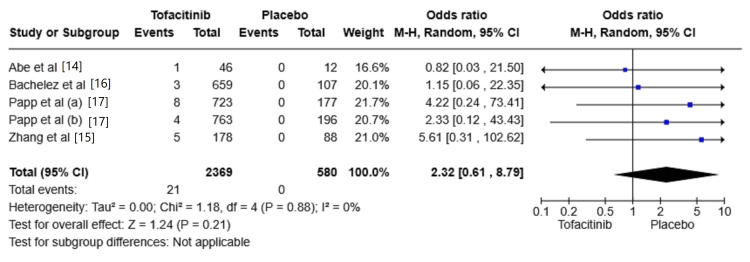
Random-effects meta-analysis from studies assessing the risk of herpes zoster (HZ). CI: Confidence Interval; df: Degrees of Freedom; OR: Odds Ratio; Z: Z-score; Chi²: Chi-square Test. The P-value is considered significant at (P < 0.05). Source: References [14-17]

Five studies reported the risk of HZ infections [14,15,16,17]. The risk of HZ infections was increased in the Tofacitinib group compared with placebo (OR: 2.32, 95% CI: 0.61-8.79; I2 = 0%). However, the difference was not significant between the two groups (P = 0.21) (Figure 3).

The risk of URTIs

**Figure 4:**
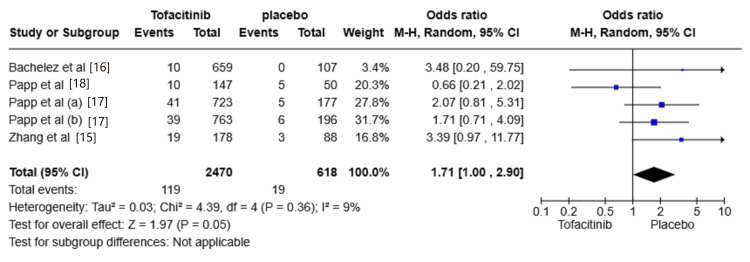
Random-effects meta-analysis from studies assessing the risk of upper respiratory tract infections (URTI). CI: Confidence Interval; df: Degrees of Freedom; OR: Odds Ratio; Z: Z-score; Chi²: Chi-square Test. The P-value is considered significant at (P < 0.05). Source: References [15-18]

Five studies reported the risk of URTIs [15-18]. The risk of URTIs was increased in the Tofacitinib group compared with placebo (OR: 1.71, 95% CI: 1.00-2.9; I^2^ = 9%). However, the results were not significant. The overall effect size was 1.97 (P = 0.05) (Figure 4).

The risk of nasopharyngitis

**Figure 5:**
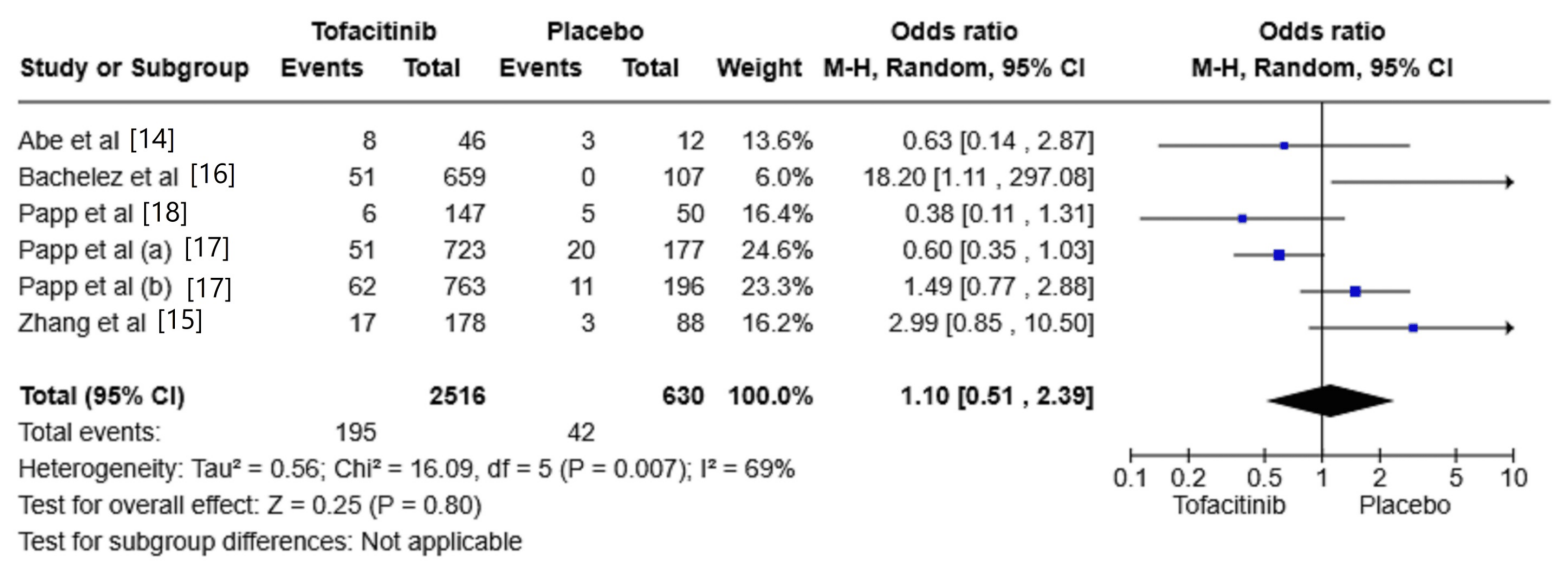
Random-effects meta-analysis from studies assessing the risk of nasopharyngitis. CI: Confidence Interval; df: Degrees of Freedom; OR: Odds Ratio; Z: Z-score; Chi²: Chi-square Test. The P-value is considered significant at (P < 0.05). Source: References [14-18]

All the included studies reported cases of nasopharyngitis [14-18]. The risk of nasopharyngitis was increased in the Tofacitinib group compared with placebo (OR: 1.10, 95% CI: 0.51-2.39; I^2^ = 69%). However, the overall effect size was insignificant at 0.25 (P = 0.80) (Figure 5).

The risk of SAEs

**Figure 6:**
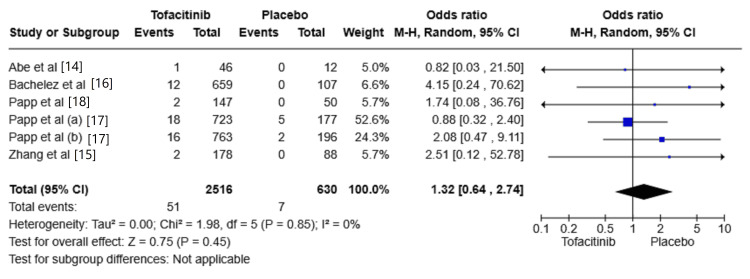
Random-effects meta-analysis from studies assessing the risk of serious adverse events (SAEs). CI: Confidence Interval; df: Degrees of Freedom; OR: Odds Ratio; Z: Z-score; Chi²: Chi-square Test. The P-value is considered significant at (P < 0.05). Source: References [14-18]

All included studies reported SAE [14,15,16,17,18]. The risk of SAE was increased in the Tofacitinib group compared with placebo (OR: 1.32, 95% CI: 0.64-2.74; I² = 0%); however, the results showed no significant difference between the two groups (P = 0.45) (Figure 6).

ROB assessment

**Figure 7:**
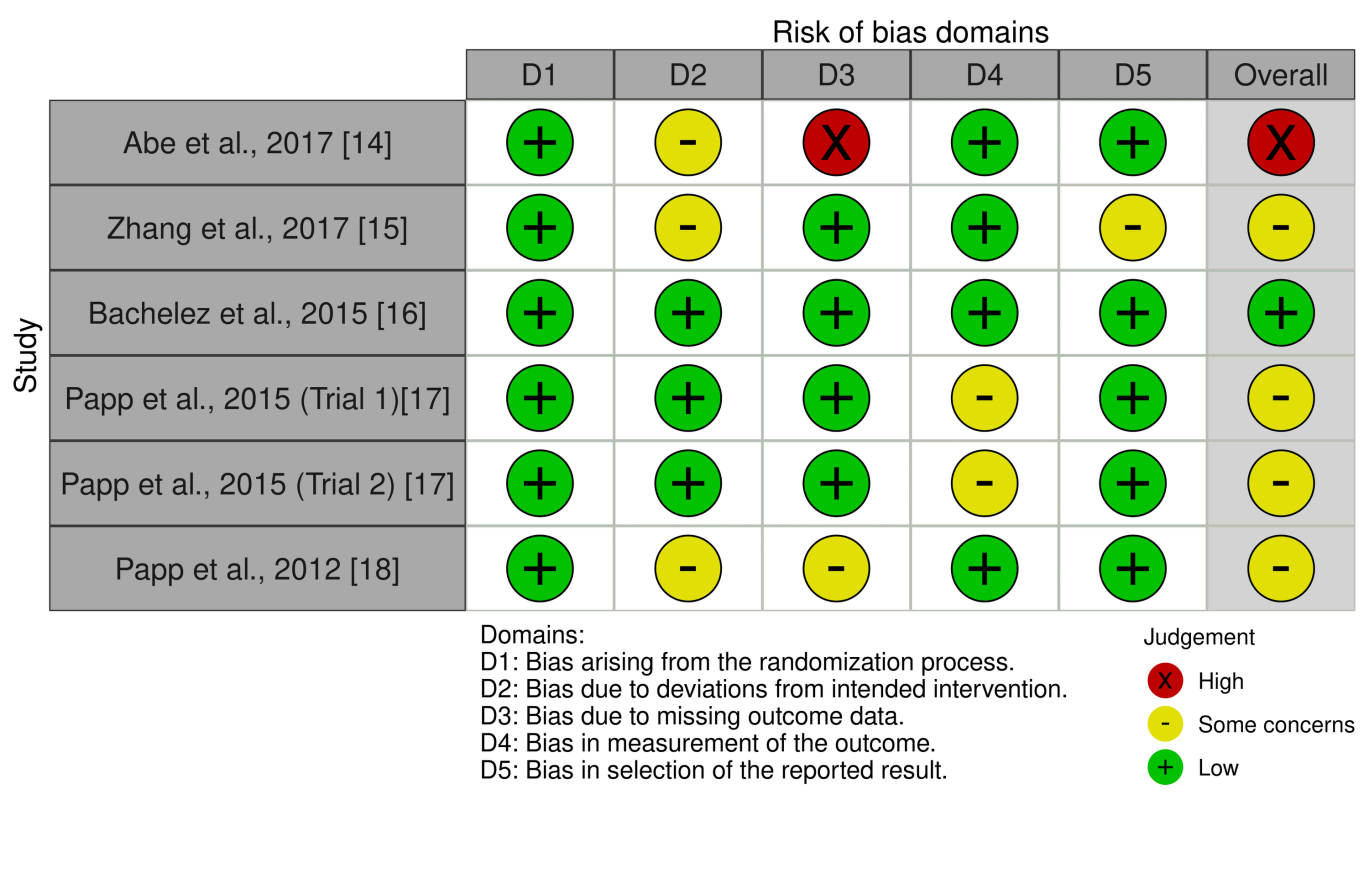
Risk of bias assessment. Source: References [14-18]

**Figure 8:**
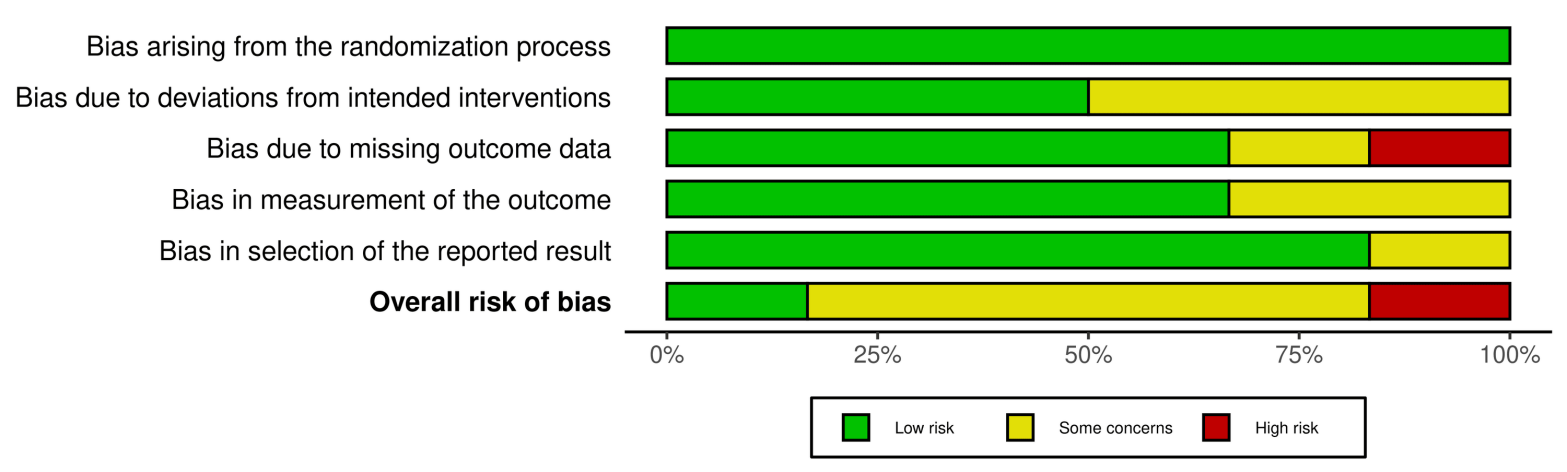
Risk of bias summary. Source: References [14-18]

The ROB was assessed using the ROB2 tool for all included trials. The summary of the ROB in each domain, as well as the overall risk, is provided in Figures 7-8. The ROB regarding randomization was low in all included studies [14-18]. Regarding the deviation from the intended intervention, three studies showed some concerns [14,15,18]. In addition, one study showed a high ROB in missing data [14], and one study had some concerns [5]. The measurement of outcomes showed low ROB except in Papp KA et al. (2015) (trials a and b), where they had some concerns [17]. The risk of selective reporting of outcomes was low in all trials but revealed some concerns in the Zhang J et al. (2017) study [15].

Discussion

This systematic review and meta-analysis aimed to assess the safety of Tofacitinib in patients with moderate-to-severe psoriasis. The incidence of serious infections, SAEs, HZ infections, URTIs, and nasopharyngitis were measured and evaluated through the meta-analysis.

Incidence of serious infections and SAEs

The analysis revealed that while the risk of serious infections was higher in the Tofacitinib group compared to the placebo, the difference was not statistically significant (OR: 0.73, 95% CI: 0.22-2.46; P = 0.62). Similarly, the risk of SAEs was also higher in the Tofacitinib group, yet it did not reach statistical significance (OR: 1.32, 95% CI: 0.64-2.74; P = 0.45). Based on these findings, it may be suggested that while Tofacitinib may increase the risk of serious adverse outcomes, the evidence generated in the current studies is not conclusive enough to confirm this hypothesis.

Other studies support this finding. For instance, Chiricozzi A et al. (2015) discussed the immunosuppressive characteristics of Tofacitinib, concluding that it may become an important therapeutic option in psoriasis management. However, the study also emphasized the importance of monitoring patients, especially those with pre-existing conditions that may exacerbate infections [19]. Additionally, Galluzzo M et al. (2016) highlighted the importance of experts being aware of signs of serious events, especially in patients with a history of recurrent infections [20].

Risk of HZ infections

According to the meta-analysis, compared with placebo, Tofacitinib increased the risk of HZ infections (OR: 2.32, 95% CI: 0.61-8.79; P = 0.21). This result aligns with a study by Winthrop KL et al. (2017), who reported HZ as an adverse event following treatment of psoriasis with Tofacitinib. The immunomodulatory effects of Tofacitinib are important to its therapeutic efficacy and may increase susceptibility to viral reactivation, including HZ [21]. Although our review found the difference in HZ infection rates between the intervention and placebo groups statistically insignificant, the observed increase in HZ cases among the intervention group remains a concern.

The pathogenesis of psoriasis is induced by STAT proteins and JAK intracellular protein tyrosine kinases including JAK1, JAK2, JAK3, and TYK2, all involved in transmitting signals of inflammatory cytokines. While JAK inhibitors act on these pathways to relieve symptoms, they modulate cytokines involved in adaptive immunity to this virus, hence rendering the patient susceptible to primary infection of HZ and virus reactivation [22]. Additionally, JAK3 and JAK1 enhance antibody production, which justifies the elevated risk of HZ infection observed with Tofacitinib, which decreases JAK3 and JAK1 proteins [23].

Risk of URTIs

Furthermore, in the current review, Tofacitinib was associated with a high risk of URTIs (P = 0.05), which is closest to statistical significance compared to other measured outcomes. This result is aligned with Azevedo and Torres (2018), who discovered that URTIs are among the most prevalent non-SAEs assessed among individuals receiving Tofacitinib [24].

Risk of nasopharyngitis

Conversely, while nasopharyngitis was more prevalent in the Tofacitinib group compared to placebo, the difference between the intervention and placebo groups was not statistically significant (P = 0.33). Similarly, in 2013, Ortiz-Ibáñez K et al. indicated that the lack of a significant increase in nasopharyngitis may be due to the assumption that such adverse events may result from environmental factors or concurrent medications rather than from the immunosuppressive effects of Tofacitinib [25].

Clinical implications

The findings of this review are important as they add to the body of literature important knowledge related to the safety profile of Tofacitinib, in addition to the rigorous patient selection and monitoring when using Tofacitinib for moderate-to-severe psoriasis. Of particular interest is the increased risk of infections; patients should be warned of this risk before therapy is initiated. The fact that the risks of serious infections and SAEs are relatively comparable makes Tofacitinib a feasible therapeutic intervention in otherwise refractory cases, with proper precautions. Several studies in this review support individualized care. Similarly, Krueger J et al. (2016) revealed that the management of psoriasis needs to be personalized for each patient based on both the extent of disease severity and health status [26].

Limitations

There are several limitations to the systematic review and meta-analysis. Firstly, with only six RCTs, the results cannot be highly generalizable. The studies were conducted in several countries, Japan, China, France, and the USA, which created variability in patients' characteristics, doctors' practices, and various environmental factors that can affect results and limit the extrapolation of these results to other populations.

## Conclusions

In conclusion, while Tofacitinib is effective against moderate-to-severe psoriasis, there are some safety concerns regarding the incidence of infections and adverse effects. Thus, managing the risk associated with such medication among vulnerable populations is crucial. Future research is needed to assess safety outcomes with long-term use of Tofacitinib in larger populations and with long-term follow-ups to provide conclusive results.
